# Inducible HSP70 Is Critical in Preventing the Aggregation and Enhancing the Processing of PMP22

**DOI:** 10.1177/1759091415569909

**Published:** 2015-02-05

**Authors:** Vinita G. Chittoor-Vinod, Sooyeon Lee, Sarah M. Judge, Lucia Notterpek

**Affiliations:** 1 Departments of Neuroscience and Neurology, College of Medicine, McKnight Brain Institute, University of Florida, Gainesville, FL, USA; 2 Department of Physical Therapy, College of Public Health & Health Professions, University of Florida, Gainesville, FL, USA

**Keywords:** chaperones, Charcot–Marie–Tooth disease, neuropathy, proteasome, Trembler-J

## Abstract

Chaperones, also called heat shock proteins (HSPs), transiently interact with proteins to aid their folding, trafficking, and degradation, thereby directly influencing the transport of newly synthesized molecules. Induction of chaperones provides a potential therapeutic approach for protein misfolding disorders, such as peripheral myelin protein 22 (PMP22)-associated peripheral neuropathies. Cytosolic aggregates of PMP22, linked with a demyelinating Schwann cell phenotype, result in suppression of proteasome activity and activation of proteostatic mechanisms, including the heat shock pathway. Although the beneficial effects of chaperones in preventing the aggregation and improving the trafficking of PMP22 have been repeatedly observed, the requirement for HSP70 in events remains elusive. In this study, we show that activation of the chaperone pathway in fibroblasts from PMP22 duplication-associated Charcot–Marie–Tooth disease type 1A patient with an FDA-approved small molecule increases HSP70 expression and attenuates proteasome dysfunction. Using cells from an HSP70.1/3^−/−^ (inducible HSP70) mouse model, we demonstrate that under proteotoxic stress, this chaperone is critical in preventing the aggregation of PMP22, and this effect is aided by macroautophagy. When examined at steady-state, HSP70 appears to play a minor role in the trafficking of wild-type-PMP22, while it is crucial for preventing the buildup of the aggregation-prone Trembler-J-PMP22. HSP70 aids the processing of Trembler-J-PMP22 through the Golgi and its delivery to lysosomes via Rab7-positive vesicles. Together, these results demonstrate a key role for inducible HSP70 in aiding the processing and hindering the accumulation of misfolded PMP22, which in turn alleviates proteotoxicity within the cells.

## Introduction

Protein misfolding and aggregation are common features in a number of neurodegenerative disorders, cumulatively referred to as proteinopathies. In the peripheral nervous system (PNS), protein aggregate formation within Schwann cells has been observed with misexpression of peripheral myelin protein 22 PMP22; (Nishimura et al., 1996; Hanemann et al., 2000; Isaacs et al., 2002; Fortun et al., 2003, 2006). PMP22 is a tetraspan hydrophobic protein, associated with a heterogeneous group of sensory and motor neuropathies, including Charcot–Marie–Tooth disease type 1A (CMT1A; Li et al., 2013). The majority of the CMT1A cases is linked with a 1.5 Mb duplication on the short arm of chromosome 17, comprising the intact PMP22 gene (Lupski et al., 1991). A smaller percentage of CMT1A patients carry a point mutation in PMP22, such as the leucine to proline (L16P) amino acid substitution, modeled by the Trembler-J (TrJ) mouse (Valentijn et al., 1992). PMP22 is synthesized predominantly by myelinating Schwann cells, but it is also expressed in nonneural cell types such as fibroblasts, endothelia, and epithelia (Manfioletti et al., 1990; Baechner et al., 1995; Notterpek et al., 2001). In normal myelinating and nonmyelinating Schwann cells, approximately 20% of the newly synthesized PMP22 acquires complex glycosylation while the remaining ∼80% is targeted for degradation by the proteasome, through endoplasmic reticulum-associated degradation (ERAD; Pareek et al., 1997). In the event of PMP22 misexpression, the amount of protein retro-translocated to the cytoplasm for degradation appears to exceed the capacity of the proteasome, resulting in PMP22 aggregate formation and a reduction in overall proteasome activity (Isaacs et al., 2002; Fortun et al., 2005, 2006). The decrease in proteasome activity is likely to promote further protein aggregation, negatively impact cellular proteostasis, and result in Schwann cell dysfunction (Kopito and Sitia, 2000; Fortun et al., 2005).

Cytosolic aggregates of PMP22 recruit heat shock proteins (HSPs) and constituents of the autophagy-lysosomal pathway, likely as an attempt to reduce the aggregate burden (Fortun et al., 2003, 2006, 2007). The chaperone or heat shock (HS) pathway is a stress-response mechanism that involves coordinated synthesis of several chaperones, also called HSPs (Morimoto, 2011). Chaperones are known to aid in the folding of newly synthesized proteins and have roles in the disaggregation and degradation of persistently misfolded proteins (Hipp et al., 2014). Elevated levels of HSPs, including HSP70, HSP40, and αB-crystallin, have been observed in *in vivo* and *in vitro* models of demyelinating neuropathies, and chaperones were shown to colocalize with PMP22 aggregates (Ryan et al., 2002; Fortun et al., 2003). Activation of the chaperone pathway by dietary modulation, HS or pharmacological small molecule therapeutics, led to a reduction in PMP22 aggregates and an improvement in the myelination capacity of neuropathic Schwann cells (Fortun et al., 2007; Rangaraju et al., 2008; Madorsky et al., 2009). In each of these paradigms, enhanced trafficking of PMP22 was associated with an increase in the levels of HSP70, which suggests a potential linkage between the two proteins. This hypothesis is supported by the discovery of a more severe neuropathic phenotype when TrJ mice were crossed with HSP70-deficient mice (Okamoto et al., 2013). Additional evidence for the involvement of HSP70 in peripheral nerve biology is signified by the attenuation of neuropathic phenotype by KU32, a synthetic HSP90 inhibitor that upregulates HSP70, in diabetic mice (Ma et al., 2014).

The stress-inducible form of HSP70 is the most abundant chaperone involved in different aspects of the proteostasis network (Saibil, 2013). HSP70 aids in the folding of proteins with its cochaperone, HSP40, and is involved in the disaggregation of misfolded proteins, in collaboration with HSP104/110 (Lee et al., 2013; Song et al., 2013). HSP70 also plays crucial roles in proteasomal or autophagic degradation of terminally misfolded proteins, through CHIP (Carboxyl-terminus of HSP70 interacting protein)-dependent mechanisms (Kettern et al., 2010). The ability of HSP70 to recognize and bind misfolded proteins is utilized in targeting the cargo to cellular degradative pathways. For example, HSP70 has been shown to reduce protein aggregation and alleviate disease phenotypes in various neurodegenerative disorders, including Alzheimer’s disease (Bobkova et al., 2014), amyotrophic lateral sclerosis (Gifondorwa et al., 2012), and tauopathies (Jinwal et al., 2013).

Although the beneficial effects of enhanced chaperone-response in reducing the aggregation of PMP22 in disease paradigms have been reported, the requirement for HSP70 in this event remains unknown. Utilizing a number of distinct cell culture models, including embryonic fibroblasts from HSP70.1/3-deficient mice (Hunt et al., 2004), we examined the contribution of HSP70 to the reduction of PMP22 aggregates. In cells from a CMT1A patient, pharmacological enhancement of HSP70 attenuates proteasome dysfunction, while this benefit is lost when similar experiments are performed in cells from HSP70-deficient mice. Our results indicate that under basal conditions, HSP70 is not essential for the processing of PMP22; however, upon proteotoxic stress, the absence of this chaperone exacerbates the aggregation of PMP22.

## Materials and Methods

### Cell Culture Models

Dermal fibroblasts from a 51-year-old CMT1A patient (GM05165) were purchased from the Coriell Institute (Camden, NJ) and maintained in minimum essential media (MEM) supplemented with nonessential amino acids, 15% to 20% fetal calf serum (FCS; Hyclone, Logan, UT), and 20 mM L-glutamine (Gibco). Per Coriell, multiplex ligation-dependent probe amplification was used to confirm the duplication (17 p) of the PMP22 gene in this patient and no mutations in the myelin protein zero (MPZ) gene. Fibroblasts from a 56-year-old nonneuropathic control individual were obtained under an IRB-approved protocol by Dr. Guang-Bin Xia (Department of Neurology, University of Florida) and provided to us for the described studies. Human fibroblast cultures were expanded once per week by dissociation with 0.05% trypsin and used for experiments under 10 passages. Mouse embryonic fibroblasts (MEFs) from wild-type (Wt) and HSP70.1/3^−/−^ mice (Hunt et al., 2004) were established from E13 embryos as described (Takahashi and Yamanaka, 2006). The use of animals for these studies was approved by University of Florida Institutional Animal Care and Use Committee (IACUC). Cultures were maintained in Dulbecco’s MEM (DMEM) with 10% FCS and used for experiments under six passages.

### Pharmacologic Treatment Paradigms

For activation of the HS pathway in human and mouse fibroblasts, an established HSP90 inhibitor, BIIB021 (Selleckchem, Houston, TX), was used at 100 nM concentration (Lundgren et al., 2009). To inhibit the proteasome, Wt and HSP70.1/3^−/−^ MEFs were plated in 2% FCS and allowed to proliferate for 24 hr. Following this, the cultures were treated with 30 μM lactacystin (Lc) (Biomol Research Laboratories, PA) or its vehicle dimethyl sulfoxide (DMSO; Sigma, St. Louis, MO), for 16 hr in 0% FCS (Notterpek et al., 1999). Treatment conditions were selected after testing multiple doses and paradigms to achieve aggregate count in 50% of the cells (Fortun et al., 2007). This experimental model allowed us to assess the effects of BIIB021 and 3-methyladenine (3-MA; Sigma, St. Louis, MO) on aggregate formation. The compound 3-MA is a known inhibitor of autophagy through class III phosphatidylinositol 3-kinases when used at 4 mM for 16 hr (Wu et al., 2010).

### Cell Transfection Experiments

For experiments with the human fibroblasts, 4 × 10^6^ cells were transfected with either Ub-G76V-green fluorescent protein (GFP; Dantuma et al., 2000) or pmaxGFP (Lonza) plasmids using the Amaxa Lonza Nucleofector kit (VPD-1001) on Amaxa Biosystems Nucleofector II, as per the manufacturer’s instructions. Cells were plated on poly-L-lysine-coated glass coverslips, and media were replaced after 4 hr with addition of DMSO or BIIB021 (100 nM). Twenty-four hours posttransfection, Hoechst dye (Molecular Probes, Eugene, OR) was added for 5 min to visualize the nuclei. Coverslips were mounted, and images were acquired with a SPOT digital camera (Diagnostic Instrumentals, Sterling Heights, MI) attached to a Nikon Eclipse E800 microscope (Tokyo, Japan). GFP-positive and total (Hoechst-positive) number of cells were counted in 8 to 10 random microscopic fields (0.1 mm^2^) and graphed as percentage of total. Transfection efficiency, as determined by pmaxGFP-positive cells, was at ∼54% in both Ct and CMT1A cultures. MEFs (2 × 10^6^ cells) were transfected with Wt-Myc3, TrJ-HA3 (Tobler et al., 1999), or pmax-GFP (6 µg DNA/transfection) using the Amaxa mouse/rat hepatocyte nucleofection kit (VPL-1004). Cells were plated on poly-L-lysine-coated dishes for 6 hr before media change. After 24 hr, the samples were fixed for immunocytochemistry or harvested for biochemical analyses. Electroporation efficiency was monitored using the pmaxGFP vector, and it was ∼23%, in both Wt and HSP70.1/3^−/−^ cultures.

### Measurement of 20S Proteasome Activity

The chymotrypsin-like activity of the 20S subunit of proteasome in human fibroblasts or MEFs was measured by changes in the fluorescence of amino-methyl coumarin (AMC) conjugated to the chymotrypsin peptide substrate LLVY, using a commercial kit (Chemicon; Fortun et al., 2005). After treatment with either DMSO or BIIB021 (100 nM) for 24 hr, fibroblasts were harvested and homogenized in ice-cold buffer containing 50 mM Tris-HCl (pH 7.5) and 1 mM EDTA. Homogenates were centrifuged at 16,000 *g* for 10 min at 4℃, supernatants collected, followed by determination of protein concentrations by bicinchoninic acid BCA assay. The degradation of LLVY-AMC was spectrophotometrically measured by the cleavage of AMC from LLVY. The proteasome inhibitor Lc (30 µM) was included as the control for specificity. The fluorescence readout for each sample was normalized to the concentration of protein and plotted as a measure of the 20S proteasome chymotrypsin-like activity.

### Biochemical Studies

Cells or sciatic nerves were homogenized in sodium dodecyl sulfate (SDS) gel sample buffer (62.5 mM Tris, pH 6.8, 10% glycerol, 3% SDS), containing protease and phosphatase inhibitors. The total protein concentrations were measured using the BCA assay (Pierce, Rockford, IL). Equal amounts of protein were separated on 10% or 12.5% SDS-polyacrylamide gels under reducing conditions, and proteins were transferred to nitrocellulose membranes (Bio-Rad Laboratories). Membranes were blocked in 5% nonfat milk in Tris-buffered saline with 0.05% Tween-20 (TBS-T) and incubated overnight with primary antibodies (Table 1). After washing, anti-rabbit, anti-mouse (Cell Signaling Technology Inc.), or anti-goat (Jackson ImmunoResearch) horseradish peroxidase (HRP)-linked secondary antibodies were added for 2 hr. Bound antibodies were visualized using an enhanced chemiluminescence detection kit (PerkinElmer Life Sciences). Films were digitally imaged using a GS-800 densitometer (Bio-Rad Laboratories) and were formatted for printing, using Adobe Photoshop 5.5.

**Table 1.** Primary Antibodies Used in This Study.

Species	Antigen	Source and catalog #	Dilution	
			WB	IS
Rabbit	HSP90	Cell Signaling Technology; E289	1:1000	n/a
Mouse	HSP70	StressMarq; SMC-100B	1:3000	n/a
Rabbit	HSP40	Stressgen; SPA-400	1:2000	n/a
Goat	HSP27	Santa Cruz Biotechnology, Inc.; sc-1049	1:1000	n/a
Rabbit	αB-crystallin	Stressgen; SPA-223	1:4000	n/a
Mouse	Tubulin	Sigma, St Louis, MO, USA ; T6199	1:2000	n/a
Mouse	PMP22	NeoMarkers; MS-1293-PABX	n/a	1:1000
Rabbit	Ubiquitin	Dako, Carpinteria, CA; Z0458	1:1000	1:1000
Mouse	GAPDH	Encor Biotechnology Inc.; MCA-1D4	1:10000	n/a
Rabbit	PMP22	Abcam; ab15506	1:1000	n/a
Mouse	Myc	Santa Cruz; sc-40 (9E10)	1:1000	1:500
Rabbit	Calnexin	Stressgen; SPA-860	1:1000	n/a
Rabbit	HA	Santa Cruz; sc-805 (Y-11)	1:1000	n/a
Mouse	HA	DSHB, University of Iowa	n/a	1:100
Rat	LAMP1	DSHB, University of Iowa	n/a	1:10
Rabbit	Rab7	Santa Cruz; sc-10767 (H-50)	1:1000	1:500
Rabbit	HSP70 (HSP70*)	Stressgen; SPA-812	n/a	1:1000
Mouse	Atf6	Abcam; ab11909	1:500	n/a
Rabbit	BiP	Stressgen;SPA-826	1:1000	n/a
Rabbit	CHOP	Santa Cruz; sc-793	1:1000	n/a

*Note*. WB = western blot; IS = immunostaining; n/a = nonapplicable; HSP = heat shock protein; PMP22 = peripheral myelin protein 22; CHOP = C/EBP homologous protein.

The processing of PMP22 was assessed using endoglycosidase H (EndoH) and N-glycosidase F (PNGaseF) enzymes (New England Biolabs, Beverly, MA; Pareek et al., 1997). Briefly, incubation of the lysates with EndoH or PNGaseF was performed for 17 hr at 37℃. Protein samples were then separated under reducing conditions on 12.5% polyacrylamide gels and processed for the detection of PMP22. Densitometric analyses of the data were carried out using ImageJ (NIH).

### Immunocytochemistry and Protein Aggregate Quantification

Cells on coverslips were fixed with 4% paraformaldehyde for 10 min and permeabilized with ice-cold 100% methanol (5 min) at −20℃, or 0.2% Triton X-100 (for LAMP1 and Rab7 staining) at room temperature, followed by blocking with 5% normal goat serum in phosphate-buffered saline (PBS), for 1 hr (Fortun et al., 2007). After an overnight incubation with primary antibodies (Table 1) at 4℃, samples were treated with Alexa Fluor 594 (red) or Alexa Fluor 488 (green) anti-rabbit, anti-mouse, or anti-rat secondary antibodies (Molecular Probes, Eugene, OR) for 2 hr at room temperature. Nuclei were visualized with Hoechst dye. Samples without primary antibodies were processed in parallel as negative controls. After washing in PBS, coverslips were mounted using the Prolong Antifade kit (Molecular Probes). Images were acquired with a SPOT digital camera attached to a Nikon Eclipse E800 or an Olympus DSU spinning disc confocal (Tokyo, Japan) microscope. Images were processed for printing using Photoshop 5.5 (Adobe Systems, San Jose, CA). Cells containing PMP22- and ubiquitin-positive aggregates (>1 µm in diameter), and the total number of cells (Hoechst positive), were counted in 8 to 10 random microscopic fields (0.1 mm^2^) using ImageJ software (NIH) and graphed as percentage of total cells (Fortun et al., 2007).

### Statistical Analyses

For all studies, means ± *SEM* were calculated and graphed using GraphPad Prism v5.0 software. Statistical significance was determined using the unpaired Student’s *t* test, and *p* value <.05 (*); <.01 (**); <.001 (***) were considered statistically significant.

## Results

### HS Pathway Activation Attenuates Proteasome Dysfunction in Fibroblasts From CMT1A Patient

**Figure 1.**
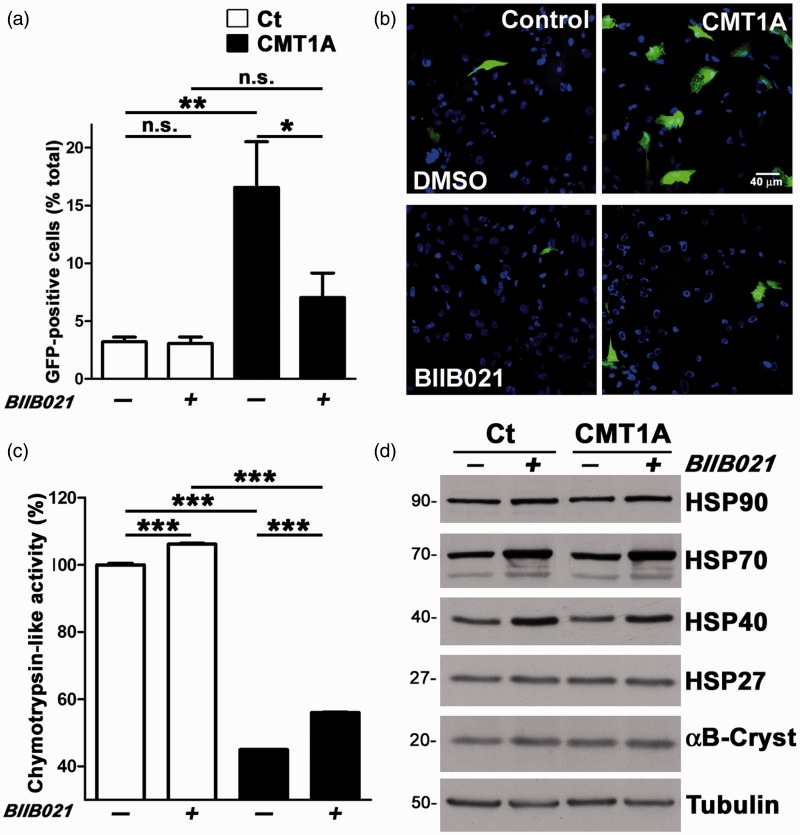
Activation of the heat shock pathway attenuates proteasome dysfunction in CMT1A patient fibroblasts. (a) Dermal fibroblasts from a control, nonneuropathic individual (Ct) and a CMT1A patient were transfected with Ub-G76V-GFP reporter and treated with either DMSO (−) or BIIB021 (+) and imaged for GFP. The number of GFP-positive cells were counted in fixed microscopic fields (0.1 mm^2^) and plotted as percentage of total (Hoechst positive) (*n* = 3 independent experiments). (b) Representative images of Ub-GFP ^+^cells in control and CMT1A cultures after treatment with DMSO or BIIB021. Hoechst dye (blue) was used to visualize nuclei. Scale bar, as shown. (c) The 20S chymotrypsin-like activity of the fibroblasts from the Ct and the CMT1A patient, after treatment with DMSO (−) or BIIB021 (+) is graphed as percentage of DMSO-treated Ct cultures (*n* = 3 independent experiments). (a, c) Graphs plotted as means ± *SEM*. Unpaired Student’s *t* test, *p < .05, **p < .01, ***p < .001, n.s.; nonsignificant. (d) Steady-state levels of HSPs, in fibroblast cell lysates (15 µg/lane) after treatment with DMSO (−) or BIIB021 (+). Tubulin serves as a loading control. Molecular mass on left, in kDa. CMT1A = Charcot–Marie–Tooth disease type 1A; DMSO = dimethyl sulfoxide; HSP = heat shock protein; GFP = green fluorescent protein.

The presence of PMP22 aggregates in peripheral nerves of neuropathic mice is associated with impaired proteasome activity, a phenotype that is mitigated by induction of chaperones (Fortun et al., 2005, 2006; Rangaraju et al., 2008). To examine whether this relationship is maintained in cells from CMT1A patients, we determined proteasome activity in human fibroblasts with and without the induction of chaperones (Figure 1(a) to (c)). Dermal fibroblasts from a 56-year-old control (Ct) and a 51-year-old CMT1A patient were transfected with the Ub-G76V-GFP reporter (Dantuma et al., 2000) and examined by fluorescence microscopy (Figure 1(a) and (b)). In this vector, a modification on the ubiquitin (Ub) moiety makes it susceptible to cotranslational proteasomal degradation and therefore Ub-GFP is only detected in cells with compromised proteasome activity (Fortun et al., 2007). Eight hours posttransfection, the cultures were treated with either DMSO (−) or the HSP90 inhibitor, BIIB021 (+) (100 nM, 24 hr) (Lundgren et al., 2009) and assessed for GFP fluorescence (Figure 1(a) and (b)). Upon quantification, we found significantly higher percentage of GFP-positive cells in DMSO-treated CMT1A cultures as compared with nonneuropathic Ct, even though transfection efficiency was only at ∼54%. Notably, treatment with the HSP90 inhibitor significantly reduced the number of GFP-positive cells in the neuropathic cultures (Figure 1(a) and (b)). In fact, the percentages of GFP-positive cells in control and CMT1A cultures after the BIIB021 compound treatment are comparable.

To substantiate the finding with the Ub-G76V-GFP reporter, next we determined the chymotrypsin-like activity of the 20S proteasome within the same cultures using a commercial assay (Figure 1(c)). Again, control and CMT1A human fibroblasts were treated with either BIIB021 (+) or DMSO (−), and the chymotrypsin-like 20S activity was graphed as the percentage of DMSO-treated control cells. As shown in Figure 1(c), the baseline 20S activity is significantly lower in CMT1A cells as compared with control. Upon treatment with BIIB021, proteasome activity similarly increases in both genotypes. While the improvement in the CMT1A samples is only about 10% to 15%, the effect is highly significant and reproducible across independent cultures. The BIIB021-associated improvement in chymotrypsin-like activity in CMT1A fibroblasts correlates with the augmented proteasomal degradation of Ub-G76V-GFP (Figure 1(a) and (b)). To confirm the bioactivity of BIIB021 in our study paradigm, we assessed the levels of applicable chaperones within whole cell lysates by western blots (Figure 1(d)). The levels of HSP70 are increased prominently in both cell types upon BIIB021 exposure (+), as compared with their respective DMSO-treated controls (−). The expression of HSP40, a cochaperone for HSP70 functions (Fan et al., 2003), is also elevated in BIIB021-treated samples, while the levels of HSP90, HSP27, and αB-Crystallin (αB-Cryst) remain steady. These results indicate that activation of the HS pathway, via inhibition of HSP90, attenuates proteasome dysfunction in fibroblasts from a CMT1A patient, possibly through an HSP70-assisted mechanism.

### HSP70 Is Critical in Preventing the Aggregation of PMP22 Under Proteotoxic Stress

**Figure 2. fig2-1759091415569909:**
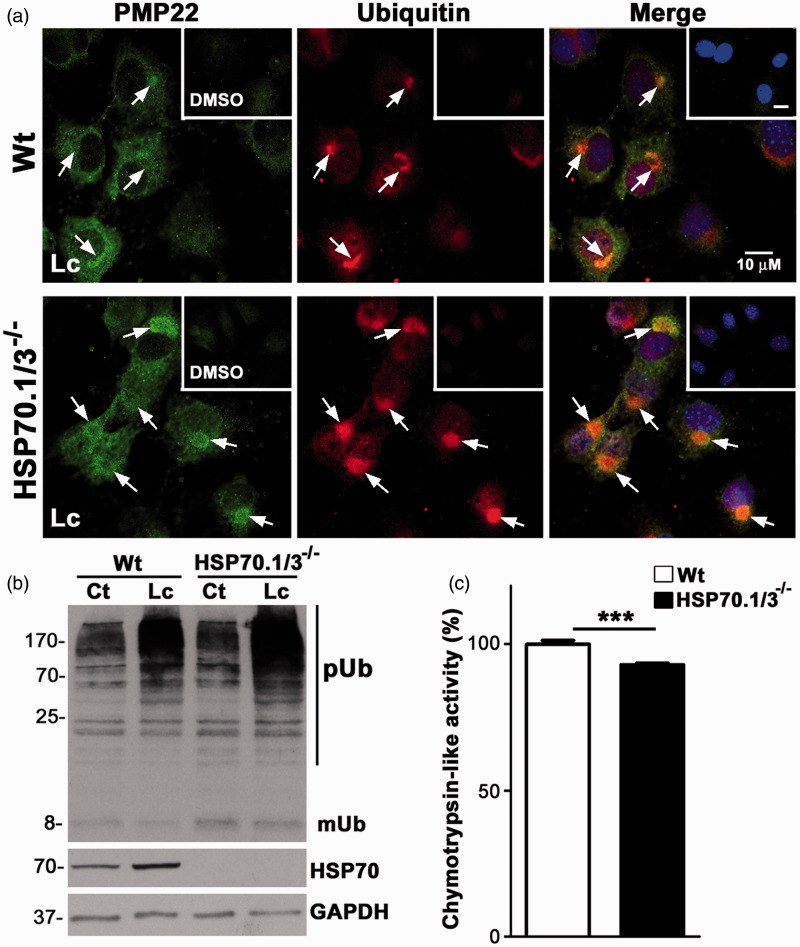
HSP70 hinders the aggregation of PMP22 in MEFs. (a) Double immunostaining of Wt and HSP70.1/3^−/−^ MEFs with anti-PMP22 (green) and anti-ubiquitin (red) antibodies, after 16 hr treatment with lactacystin (Lc) or DMSO (insets). Arrows point to PMP22- and Ub-positive protein aggregates. Nuclei are stained with Hoechst dye (blue). Scale bars, 10 µm. (b) Whole cell lysates (15 µg/lane) of Wt and HSP70.1/3^−/−^ MEFs after treatment with DMSO (Ct) or Lc were analyzed for the levels of ubiquitinated substrates and inducible HSP70. Nonconjugated mono-ubiquitin is designated as mUb. GAPDH is shown as a protein loading control. Molecular mass on left, in kDa. (c) The 20S chymotrypsin-like activity of Wt and HSP70.1/3^−/−^ MEFs is graphed as means ± SEM (*n* = 3 independent cultures). Unpaired Student’s *t* test, ***p < .001. PMP22 = peripheral myelin protein 22; DMSO = dimethyl sulfoxide; HSP = heat shock protein; MEFs = Mouse embryonic fibroblasts; pUb = poly-ubiquitinated; mUb = mono-ubiquitin; GAPDH = Glyceraldehyde 3-phosphate dehydrogenase.

To evaluate the involvement of HSP70 in mediating the positive effects of BIIB021 on relieving proteasome impairment and possibly modulating the processing of PMP22, we used cells from a mouse model in which both inducible forms of HSP70, HSP70.1 and HSP70.3 (HSP70.1/3^−/−^), are deleted (Hunt et al., 2004). These mice, however, retain intact copies of the constitutive form of this chaperone, HSC70, and are viable. We established MEFs from Wt and HSP70.1/3^−/−^ animals and examined the processing of PMP22 upon proteasome inhibition, using established paradigms (Notterpek et al., 1999; Figure 2). Upon exposure of the cells to Lc (30 μM, 16 hr), PMP22-like immunoreactivity becomes prominent as compared with DMSO control (Figure 2(a), inset), and the protein is detected in cytosolic aggregates (Figure 2(a), arrows). Essentially all PMP22-containing protein aggregates are reactive for ubiquitin, which is in agreement with our previous studies in Schwann cells (Notterpek et al., 1999). PMP22-positive aggregates are even more pronounced and appear larger in size when the proteasome is inhibited in cells from the HSP70.1/3^−/−^ mice (Figure 2(a), bottom panel). For quantification, we established a >1 µm diameter size criterion (Fortun et al., 2007), which yielded ∼16% PMP22-aggregate-containing cells in Wt cultures, compared with ∼24% in the absence of HSP70.1/3 (see Figure 4(a) for graph). Quantification of ubiquitin-positive >1 µm aggregates within the same cultures also reveals a significant increase when HSP70 is absent (see Figure 4(b) for graph).

To corroborate the immunocytochemical studies, we analyzed the levels of poly-ubiquitinated proteasome substrates within the same culture paradigms (Figure 2(b)). In accordance with fewer Ub-reactive aggregates (Figure 2(a)), we detected lower levels of poly-ubiquitinated (pUb) substrates and mono-ubiquitin (mUb) in Lc-treated Wt MEFs, as compared with HSP70.1/3^−/−^ (Figure 2(b)). While the levels of pUb-substrates increase in both genotypes upon proteasome inhibition (Figure 2(b)), this increment is less pronounced in Wt cultures. As an expected response to the proteotoxic stress, an increase in HSP70 levels is observed in Wt MEFs upon Lc-treatment, while the HSP70.1/3^−/−^ cells are unresponsive (Figure 2(b)). These results indicate that the presence of HSP70 hinders the aggregation of PMP22 and of other proteasome substrates.

As the baseline pUb levels in DMSO-treated cultures suggest a mild impairment in proteasome function in the absence of HSP70.1/3, we determined the 20S chymotrypsin-like activity in lysates of Wt and HSP70.1/3^−/−^ MEFs (Figure 2(c)). Using the sensitive LLVY-AMC substrate-based fluorescence assay, we found a small (∼7%), but highly significant decrease in proteasome activity in the absence of HSP70. Together these data indicate that in the absence of HSP70.1/3, there is a mild proteotoxic stress within the cells, which is accentuated when proteasome activity is further inhibited by pharmacological manipulation (Lc).

**Figure 3. fig3-1759091415569909:**
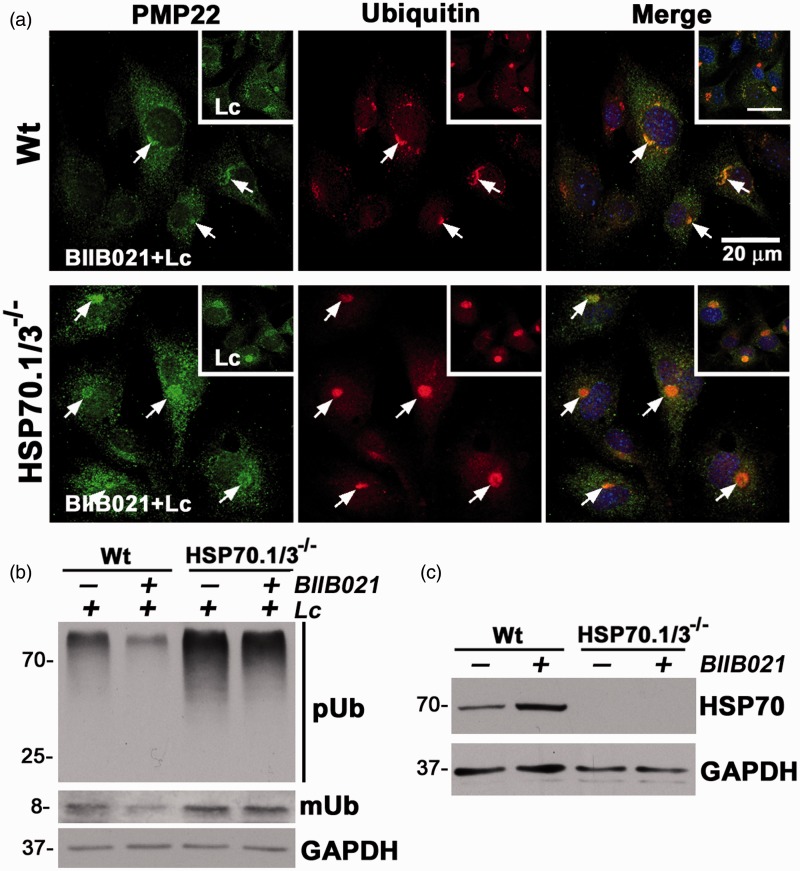
Pharmacological induction of chaperones suppresses PMP22 aggregation in Wt, but not in HSP70.1/3^−/−^ MEFs. (a) Representative images of Wt and HSP70.1/3^−/−^ MEFs after 16 hr treatment with Lc and pretreatment with BIIB021 or DMSO (insets) for 8 hr. Arrows indicate the PMP22- (green) and Ub- (red) positive aggregates. Hoechst dye (blue) is used to visualize the nuclei. Scale bars, 20 µm. (b) The levels of poly-ubiquitinated substrates (pUb) were analyzed in whole cell lysates (15 µg/lane) of Wt and HSP70.1/3^−/−^ MEFs after pretreatment with BIIB021 (+) or DMSO (−), followed by Lc treatment. The nonconjugated mUb is also marked. (c) Whole cell lysates (20 µg/lane) of Wt and HSP70.1/3^−/−^ MEFs, treated with BIIB021 (+) or DMSO (−) for 8 hr were analyzed for the levels of inducible HSP70. (b, c) GAPDH serves as a loading control. Molecular mass in kDa, on left. PMP22 = peripheral myelin protein 22; MEFs = Mouse embryonic fibroblasts; HSP = heat shock protein; DMSO = dimethyl sulfoxide; mUb = mono-ubiquitin; GAPDH = Glyceraldehyde 3-phosphate dehydrogenase.

**Figure 4.**
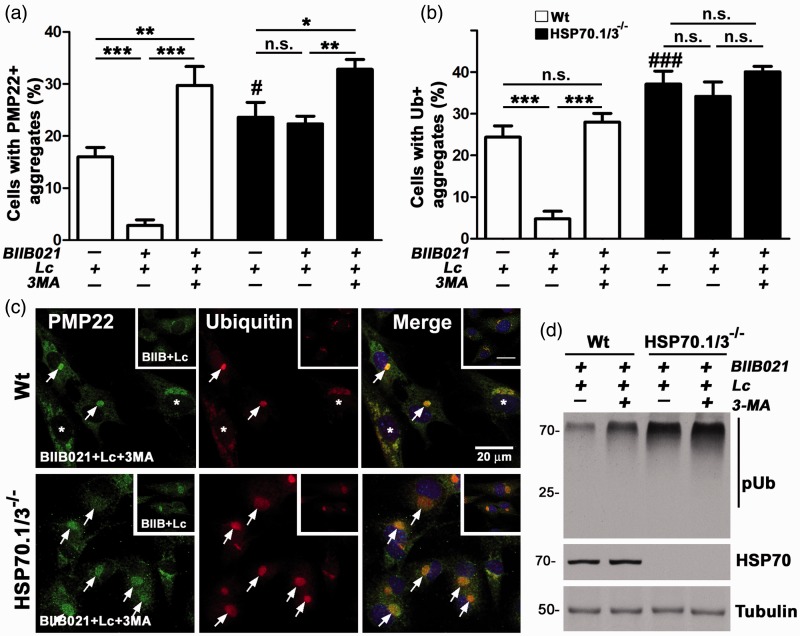
Autophagy aids in the reduction of PMP22 aggregates by HSP70. (a) The percentage of PMP22- and (b) Ub-positive aggregate-containing cells after the indicated treatments were counted and graphed. Data are shown as means ± *SEM*. Unpaired Student’s *t* test, ###*p* < .001, #*p* < .05 (across genotypes between Lc-treated cultures), *p < .05, **p < .01, ***p < .001, n.s.; nonsignificant (*n* = 3 independent experiments). (c) PMP22 (green) and Ub (red)-reactive protein aggregates in Wt and HSP70.1/3^−/−^ MEFs after sequential treatment with BIIB021 and Lc alone (insets, BIIB + Lc), or in combination with 3-MA (BIIB021 + Lc + 3MA). Arrows point to PMP22 and Ub-positive aggregates. Asterisks mark cells with dispersed aggregates. Hoechst dye (blue) was used to visualize nuclei. Scale bars, 20 µm. (d) Whole cell lysates (15 µg/lane) of Wt and HSP70.1/3^−/−^ MEFs were analyzed for levels of pUb-substrates and inducible HSP70. Tubulin serves as a protein loading control. Molecular mass in kDa, on left. PMP22 = peripheral myelin protein 22; MEFs = Mouse embryonic fibroblasts; HSP = heat shock protein; pUb = poly-ubiquitinated.

Preconditioning of Schwann cells with HS was shown to hinder the aggregation of PMP22 (Fortun et al., 2007); however, the contribution of HSP70.1/3 to this benefit has not been investigated. Using BIIB021 to pharmacologically induce HSPs in cells from Wt and HSP70.1/3^−/−^ mice, we compared protein aggregation between the cultures after Lc treatment (Figure 3). Wt and HSP70.1/3-deficient MEFs were pretreated either with DMSO (−) or BIIB021 (+) (100 nM) for 8 hr, followed by incubation with Lc (30 µM) for 16 hr to induce proteotoxic stress. As in Figure 2, we immunostained the cultures for PMP22 and Ub and calculated the number of cells with PMP22 and Ub-positive aggregates with a diameter >1 µm (see Figure 4(a) and (b) for graph). The representative micrographs in Figure 3(a) show the morphology of the aggregates after induction of the HS pathway. While there are aggregates in some of the Wt cells, they appear less compact and are smaller in size (fall below the >1 µm criteria) (Figure 3(a), arrows in upper panel), as compared with Lc alone (Figure 2(a)). Indeed, quantification of the percentage of cells with protein aggregates reveal that in response to BIIB021 pretreatment fewer Wt cells contain aggregates, as compared with DMSO (Figure 4(a) and (b)). In Wt cells, this effect is highly significant (***p < .001) for both PMP22 and Ub-positive aggregates. On the other hand, MEFs from the HSP70.1/3^−/−^ mice did not benefit from the BIIB021 pretreatment (Figure 3(b)), as the percentages of cells with PMP22 or Ub-positive aggregates are essentially unchanged (Figure 4(a) and (b)).

As a biochemical assessment of protein aggregation, we determined the levels of pUb-substrates within the same cultures by western blots (Figure 3(b)). As shown on the representative blot, the steady-state levels of Ub-containing proteins are decreased in BIIB021 (+) pretreated Wt MEFs, as compared with DMSO (−). In comparison, the difference in the levels of pUb-reactive proteins between DMSO and BIIB021 pretreated HSP70.1/3^−/−^ MEFs is small. To confirm the induction of HSP70 by the 8 hr long BIIB021 pretreatment, representative cultures were lysed for western blots with HSP70 antibodies. As shown in Figure 3(c), Wt cells respond appropriately to BIIB021 by increasing HSP70 levels, while knockout cells are unable to respond. Together, these results show that the beneficial effects of HS pathway in hindering the aggregation of PMP22 and other Ub-positive proteasome substrates require HSP70.1/3.

### Autophagy Contributes to the Removal of PMP22 Aggregates in the Presence and Absence of HSP70

Undegraded proteasome substrates, including PMP22, can be cleared from cells through autophagy (Fortun et al., 2007; Lamark and Johansen, 2010). As HSP70 has been shown to aid in proteasomal and autophagic degradation of persistently misfolded proteins (Kettern et al., 2010), we next examined the contribution of autophagy in PMP22 aggregate removal in response to BIIB021 pretreatment (Figure 4). Subsequent to induction of the HS response, as in Figure 3, we blocked autophagy with 3-MA (3-methyladenine, 4 mM) (Wu et al., 2010) in combination with proteasome inhibition (Lc; 30 μM), and immunostained the cells for PMP22 and Ub, as above (Figures 2 and 3). In Wt cells, where PMP22- and Ub-aggregates were previously suppressed by BIIB021 pretreatment, the inclusion of 3-MA led to the retention of these proteasome substrates (Figure 4(a) and (b)). Analyses of data from independent Wt cultures show a significant (***p < .001) increase in the percentage of cells with PMP22- and Ub-positive aggregates with inclusion of 3-MA (BIIB021 + Lc + 3MA), as compared with BIIB021 + Lc alone. This increase is more pronounced for PMP22 than for ubiquitin, as inhibition of autophagy boosts the number of cells with PMP22-containing protein aggregates above Lc alone (Figure 4(a)). A basal role for autophagy in reducing the build-up of misfolded PMP22, independent of HSP70, is supported by the findings in the HSP70.1/3^−/−^ MEFs, where we identified an increase in the percentage of aggregate-containing cells when 3-MA is added (Figure 4(a)). As PMP22-containing aggregates comprise a fraction of undegraded Ub-positive proteasome substrates (Fortun et al; 2005), the effects of 3-MA are similar when Ub-reactive aggregates are quantified (Figure 4(b)).

Representative confocal micrographs of the compound-treated cells are shown in Figure 4(c) and reveal pronounced, well-defined PMP22- and Ub-positive aggregates in the HSP70.1/3^−/−^ MEFs. While the Wt cells also contain aggregated proteins, in some of the cells they appear less compact and are dispersed throughout the cytosol (Figure 4(c), asterisks in upper panel). To corroborate the immunocytochemical data, we performed western blots on cell lysates with anti-ubiquitin antibodies (Figure 4(d)). As shown on the blot, inhibition of autophagy with 3-MA leads to the accumulation of pUb-substrates in Wt MEFs, even under elevated chaperone expression. In HSP70.1/3^−/−^ cells, the increase in ubiquitin-reactive proteins in response to 3-MA is less pronounced (Figure 4(d)), which agrees with the morphological studies (Figure 4(b)). Together, these results indicate that HSP70.1/3 contributes to the removal of misfolded PMP22 by autophagy.

### HSP70 Facilitates the Processing of Mutant TrJ-PMP22

**Figure 5.**
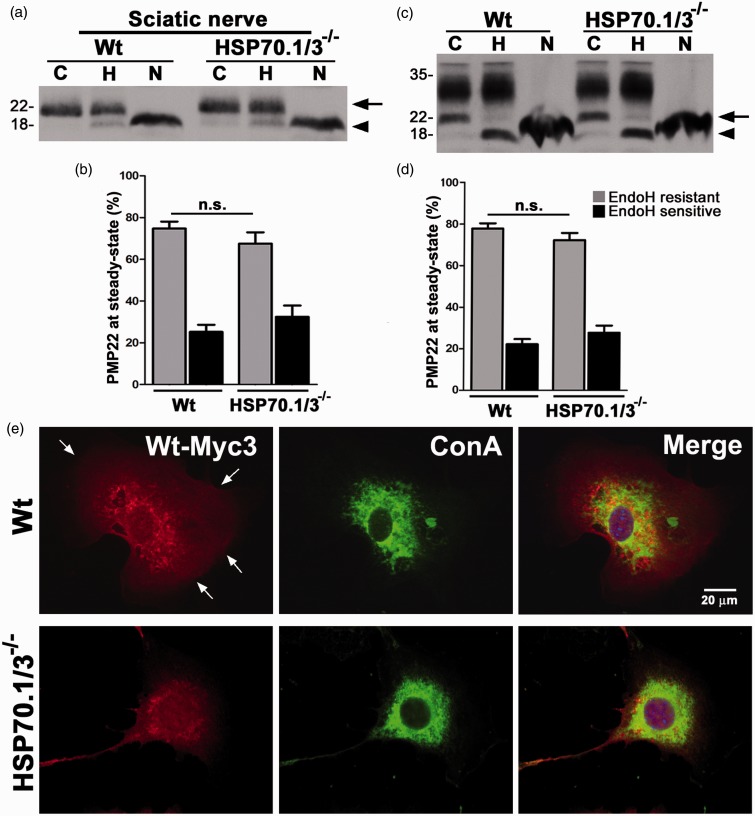
Inducible HSP70 is not critical for Wt-PMP22 trafficking. (a) Lysates of sciatic nerves (5 µg/lane) from Wt and HSP70.1/3^−/−^ mice were incubated with no enzyme (Control, C), Endoglycosidase H (EndoH, H), or N-Glycosidase F (PNGaseF, N). (b) Quantification of EndoH-sensitive and EndoH-resistant PMP22 fractions in sciatic nerve lysates from independent experiments (*n* = 3). (c) Whole cell lysates (35 µg/lane) of Wt and HSP70.1/3^−/−^ MEFs, transfected with Wt-Myc3, were incubated with no enzyme (C), EndoH (H) or PNGaseF (N) and probed for myc. (a, c) Arrows point to the EndoH- resistant fractions while arrowheads mark the EndoH-sensitive fractions. Molecular mass in kDa, on left. (d) Quantification of EndoH-sensitive and EndoH-resistant Wt-Myc3 fractions from independent experiments (*n* = 3). (b, d) Graphs plotted as means ± *SEM*. Unpaired Student’s *t* test, n.s. = nonsignificant. (e) MEFs transfected with Wt-Myc3 were immunostained for myc (red) and Concanavalin A (ConA, ER marker; green). Arrows point at the cell boundary. Hoechst dye (blue) was used to visualize the nuclei. Scale bar, as shown. PMP22 = peripheral myelin protein 22; HSP = heat shock protein; MEFs = Mouse embryonic fibroblasts.

The trafficking of PMP22 through the secretory pathway is enhanced by induction of the HS response, with concomitant increase in HSP70 (Rangaraju et al., 2008). Therefore, besides aiding the removal of misfolded PMP22 (Figure 4), HSP70 may facilitate the trafficking of the newly synthesized protein. To investigate the direct effect of HSP70 on PMP22 processing, sciatic nerve lysates from Wt and HSP70.1/3^−/−^ mice were incubated with endoglycosidase H (EndoH, H), or N-glycosidase F (PNGaseF, N) and processed for immunoblotting with an anti-PMP22 antibody (Figure 5(a)). EndoH cleaves the high mannose sugar residues on PMP22, which are then modified by mannosidase II in the medial-Golgi, rendering EndoH resistance to the protein (Pareek et al., 1997; Figure 5, arrows). PNGaseF on the other hand removes the N-glycan moiety from PMP22, revealing the ∼18 kDa core peptide (Figure 5, arrowheads). In whole nerve lysates from HSP70.1/3^−/−^ mice, we found a ∼9.6% decrease in the EndoH-resistant PMP22 fraction, as compared with age-matched Wt (Figure 5(b)). Due to variability among the samples, this decrease, however, is not significant and may reflect compensatory mechanisms within the nerves of the transgenic mice.

Thus, next we examined the trafficking of PMP22 in MEFs from Wt and HSP70.1/3^−/−^ mice using a previously characterized myc-tagged Wt-PMP22 construct (Wt-Myc3; Tobler et al., 1999). Twenty-four hours post transfection with Wt-Myc3, equal amounts of MEF cell lysates were incubated with endoglycosidases, as above, and blotted with an anti-myc antibody (Figure 5(c)). The exogenous myc-tagged PMP22 acquires heterogeneous glycosylation in cells (Tobler et al., 1999), resulting in a broad smear on the blot (Figure 5(c)). In accordance with the results from the nerves (Figure 5(a) and (b)), in both Wt and HSP70.1/3^−/−^ MEFs, a similar fraction of PMP22 acquires EndoH resistance (Figure 5(c), arrow). While analyses from three independent experiments detects ∼10% decrease in the EndoH-resistant PMP22 fraction in the absence of HSP70, this change is not significant (Figure 5(d)). As a follow-up to the biochemical studies (Figure 5(c) and (d)), we determined the subcellular distribution of myc-PMP22 in Wt and HSP70.1/3^−/−^ MEFs (Figure 5(e)). As shown on the micrographs, Wt-Myc3 PMP22 is detected in the plasma membrane of the normal MEFs (Figure 5(e), arrows), a localization that is not apparent in the HSP70-deficient cells. Nonetheless, we found no palpable retention of Wt-myc3 PMP22 in the ER or the Golgi (not shown), which is in agreement with the majority of the protein being resistant to EndoH digestion even in the absence of HSP70 (Figure 5(c) and (d)). It is still possible that HSP70 has a role in mediating the processing of the newly synthesized PMP22; however, our steady-state analyses might not be sensitive enough to detect it.

**Figure 6.**
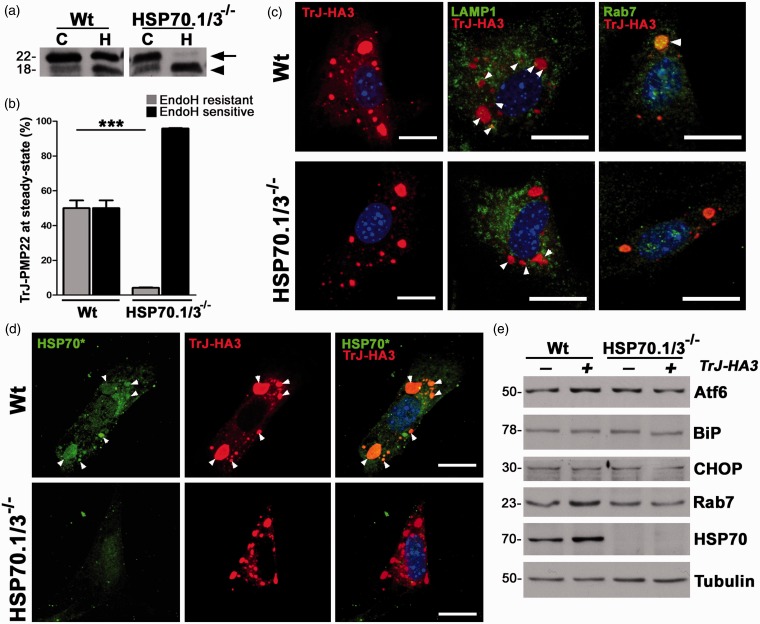
Absence of HSP70 severely impairs the processing of TrJ-PMP22. (a) Lysates from Wt and HSP70.1/3^−/−^ MEFs (35 µg/lane), transfected with TrJ-HA3, were treated with either no enzyme (C) or EndoH (H). Membranes were probed with an anti-HA antibody. Arrow points to the EndoH-resistant protein fractions while arrowhead marks the EndoH-sensitive form. (b) Quantification of the EndoH-resistant and EndoH-sensitive TrJ-HA3 fractions from (a) from independent experiments (*n* = 3). Graph plotted as means ± *SEM*. Unpaired Student’s *t* test, ***p < .001. (c) Wt (upper panel) and HSP70.1/3^−/−^ MEFs (lower panel), transfected with TrJ-HA3, were immunostained for either HA (red) alone or in combination with LAMP1 (green) or Rab7 (green). (d) TrJ-PMP22 expressing Wt and HSP70.1/3^−/−^ MEFs were probed with anti- HA (red) and anti-HSP70 antibodies. The anti-HSP70 antibody recognizes both the inducible and constitutive forms of HSP70 (HSP70*, green). (c, d) Hoechst dye (blue) was used to visualize the nuclei. Scale bars, 20 µm. Arrowheads mark areas of colocalization. (e) Lysates (30 µg/lane) of transfected MEFs were probed for the indicated proteins. Tubulin serves as a protein loading control. (a, e) Molecular mass in kDa, on left. PMP22 = peripheral myelin protein 22; HSP = heat shock protein; MEFs = Mouse embryonic fibroblasts; TrJ = Trembler-J; CHOP = C/EBP homologous protein; BiP = Binding immunoglobulin protein.

To challenge the protein homeostatic network of HSP70.1/3^−/−^ cells, we expressed the folding-destabilized, neuropathy-linked TrJ-PMP22 (Myers et al., 2008). A recent study showed potentiation of the TrJ phenotype in the absence of HSP70, implying the involvement of this chaperone in TrJ-PMP22 processing (Okamoto et al., 2013). To examine the influence of HSP70 on the trafficking of TrJ-PMP22, MEFs from Wt and HSP70.1/3^−/−^ mice were transfected with TrJ-HA3 (Tobler et al., 1999) and analyzed by western blots after incubation with EndoH (Figure 6(a)). In Wt cells, about 50% of TrJ-PMP22 acquires complex glycosylation (resistance to EndoH; Figure 6(a), arrow), while in HSP70.1/3^−/−^ MEFs, this fraction is decreased to ∼6% (Figure 6(a) and (b)). The difference in the carbohydrate modification of TrJ-PMP22 in the presence and absence of HSP70 suggests that the protein is localized in distinct subcellular compartments in Wt and HSP70.1/3^−/−^ MEFs. Therefore, next we examined the distribution of TrJ-HA3 within the same culture paradigms (Figure 6(c)). In both cell lines, TrJ-HA3 is detected in prominent cytosolic aggregates that are negative for markers of the ER (ConA) and the Golgi (VVL) (not shown). In Wt MEFs, there is a diffuse HA-like staining, which likely represents the ∼50% EndoH-resistant TrJ-HA fraction detected by the biochemical studies (Figure 6(a) and (b)).

To identify the subcellular localization of the TrJ aggregates, we costained transfected MEFs with anti-HA and anti-LAMP1 antibodies (Figure 6(c), middle panels). In both Wt and HSP70.1/3^−/−^ MEFs, LAMP1-reactive lysosomes are detected near the aggregates, yielding partial colocalization of the two markers (Figure 6(c), arrowheads). In agreement with our previous study (Notterpek et al., 1997), this data suggest involvement of the endosomal-lysosomal pathway in the processing of TrJ-PMP22. To further examine this possibility, we probed the transfected cells for Rab7, a member of the small GTPase family critical in controlling the fusion of late endosomes with lysosomes (Bucci et al., 2000). In Wt MEFs, we find a distinct colocalization of TrJ-HA3 with Rab7, while this association is absent in HSP70.1/3^−/−^ cells (Figure 6(c), right panels).

To examine the potential recruitment of HSP70 to the TrJ aggregates in Wt cells, we costained the cultures with antibodies that recognize both inducible (HSP70) and constitutive (HSC70) HSP70 (designated as HSP70*) and anti-HA (Figure 6(d)). In Wt MEFs, HSP70* is detected near and around HA-reactive aggregates (Figure 6(d), arrowheads). However, in the knockout cells, these HSP70-positive focal structures are absent, and there is only a faint HSP70-like immunoreactivity, which represents HSC70 (Figure 6(d), bottom panel). These results indicate that in the presence of HSP70, a fraction of TrJ-PMP22 is trafficked from the ER to the Golgi where it undergoes maturation and thus achieves EndoH-resistance. The mature TrJ protein is then targeted to the lysosomes via Rab7-positive endocytic vesicles. Alternatively, in the absence of HSP70, the majority of newly synthesized TrJ-PMP22 is retro-translocated from the ER to the cytosol and targeted for degradation by the proteasome and lysosomes.

In parallel with the immunocytochemical studies, we examined the potential activation of the ER-stress response when TrJ-PMP22 is expressed (Figure 6(e)). Atf6 (Activating transcription factor 6) is an ER-stress sensor that initiates the transcription of UPR (Unfolded protein response) proteins (Haze et al., 1999), including the ER-resident chaperone, BiP (Binding immunoglobulin protein; Lee, 2005). CHOP (C/EBP homologous protein) is another ER-stress sensor, regulated at the transcriptional level, which leads to apoptosis (Oyadomari and Mori, 2004). As shown on the blot, we did not detect notable changes in the levels of Atf6, BiP, or CHOP in either Wt or HSP70.1/3^−/−^ cells when TrJ-HA3 is expressed (Figure 6(e)). In accordance with the immunolabeling (Figure 6(c) and (d)), we did identify an increase in the levels of Rab7 and HSP70 in transfected Wt cells, as compared with nontransfected Wt and HSP70-deficient cultures (Figure 6(e)). Together, these studies suggest that HSP70 facilitates the removal of the aggregation-prone TrJ-PMP22 through the endocytic-lysosomal pathway.

## Discussion

**Figure 7.**
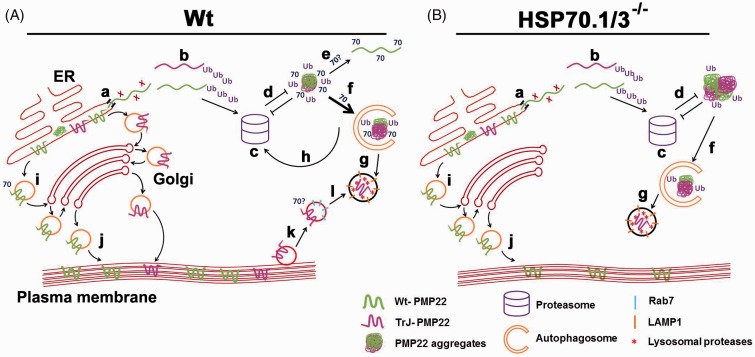
Working model of Wt and TrJ-PMP22 trafficking in the presence and absence of HSP70. (a) In normal MEFs, Wt-PMP22 and TrJ-PMP22 are trafficked out the ER (a). ‘X’ (red) represents unknown protein/s involved in the translocation. Approximately 22% of Wt-PMP22 and about half of TrJ-PMP22 are translocated (b) to the proteasome (c) for degradation. Intracellular PMP22 aggregates impair the proteasome function (d). However, the presence of HSP70 hinders aggregate formation, by increasing the solubility of cytoplasmic PMP22 (e) and facilitating the removal of misfolded protein through autophagy (f). Aggregates engulfed by autophagosomes are degraded in LAMP1-positive lysosomes (g). Fractions of Wt- and TrJ-PMP22 are processed through the Golgi (i) and inserted into the plasma membrane (j). Due to structural instability, the TrJ-PMP22 is endocytosed (k) via Rab7-positive endosomes for degradation (l) by lysosomes. (b) In case of HSP70.1/3^−/−^ cells, there is increased protein aggregate formation that can impair the proteasome function (d). In HSP70-deficient cells, Wt-PMP22 still reaches the plasma membrane (i, j), while TrJ-PMP22 fails to reach the Golgi in appreciable levels. PMP22 = peripheral myelin protein 22; HSP = heat shock protein; TrJ = Trembler-J; MEFs = Mouse embryonic fibroblasts.

Our results reveal that in a number of experimental models, including cells from a CMT1A patient, pharmacologically treated MEFs and in cell transfection paradigms, HSP70.1/3 is critical in preventing the accumulation of PMP22 aggregates (Figure 7). This effect includes the diversion of Wt-PMP22 to autophago-lysosomes under proteasome inhibition (Figures 1 [Fig fig2-1759091415569909] [Fig fig3-1759091415569909] to 4), and the removal of the aggregation-prone TrJ-PMP22 upon instability at the plasma membrane (Figure 6). While in the MEF cell model the requirement of HSP70 only becomes evident under proteotoxic stress, in fibroblasts from a CMT1A patient the expression of HSP70 is beneficial in preventing the aggregation of PMP22 and alleviating proteasome impairment. Furthermore, as the current study is limited to examination of PMP22 trafficking at steady-state, additional roles for HSP70.1/3 in mediating the kinetics of PMP22 processing cannot be ruled out.

PMP22 has a high propensity to misfold due to lack of conformational stability (Schlebach et al., 2013). Following the processing of the newly synthesized PMP22 by ^35^S-pulse labeling, we discovered that ∼80% of the protein is translocated to the cytoplasm for degradation by the proteasome (Pareek et al., 1997). Although the proteins assisting in the translocation of the unfolded PMP22 have not been identified, the transport of PMP22 to the proteasome likely occurs after its complete extraction from the ER because PMP22 aggregates are excluded from the major organelles (Notterpek et al., 1999; Fortun et al., 2006). At steady-state, this rapidly degraded pool of EndoH-sensitive PMP22 is not visible and only the accumulated, EndoH-resistant protein is detected. Working in fibroblasts, which do express endogenous PMP22 (Manfioletti et al., 1990), but lack PMP22-binding Schwann cell proteins, such as protein zero, allowed us to evaluate the involvement of HSP70 in the processing of this aggregation-prone molecule. Under compromised proteasome activity (Figures 1 [Fig fig2-1759091415569909] [Fig fig3-1759091415569909] to 4), we found that HSP70 is critical in reducing the accumulation of misfolded PMP22, partially through autophagy. In addition, HSP70 could also be involved in increasing the solubility of cytoplasmic PMP22 (Figure 7(a); Liberek et al., 2008).

Previous studies revealed that chaperones can prevent the aggregation and promote the membrane insertion of PMP22 (Ryan et al., 2002; Fortun et al., 2003, 2006, 2007); however, until now the requirement for HSP70 in this outcome was not examined. Using BIIB021, we effectively enhanced the levels of HSP70 and attenuated proteasome dysfunction in fibroblasts from a CMT1A patient (Figure 1). Apart from CMT1A, an association between proteasome dysfunction and aggregation of misfolded proteins has been reported, including in Huntington’s disease (HD; Ding et al., 2002; Verhoef et al., 2002; Bennett et al., 2005; Hipp et al., 2012). In a recent study of HD, it was shown that the aggregation-prone N-terminal huntingtin fragment itself is neither a competitive, nor a noncompetitive inhibitor of the proteasome (Hipp et al., 2012); however, one or more components of the protein quality control network may be overwhelmed by expression of the folding-impaired protein. The HSP70/40 chaperone duo was hypothesized to be a limiting factor (Hipp et al., 2012). Our observations on the attenuation of proteasome dysfunction concomitant with increased HSP70 levels in CMT1A fibroblasts support such mechanism. Elevated levels of HSP70 could aid in recruiting ubiquitin-conjugating enzymes, such as CHIP, and aid in reducing aggregate burden through autophagy (Kettern et al., 2010).

Proteotoxic stress elicited by pharmacological inhibition of the proteasome amplifies the role of inducible HSP70 in reducing PMP22 aggregation (Figure 2). We also discovered that under basal conditions, HSP70 assists proteasome function, as revealed by reduced chymotrypsin-like 20S activity in HSP70.1/3^−/−^ MEFs. However, the reduced proteasome activity in HSP70.1/3^−/−^ cells does not result in any visible Ub/PMP22-positive aggregates at baseline (Figure 2(b), insets), implying an underlying compensatory mechanism. Upon stress, however, the presence of HSP70 hinders the formation of PMP22/Ub^+^ aggregates, and this effect is enhanced with elevating HSP70 levels by BIIB021 (Figure 3). The lack of benefit of BIIB021 treatment in HSP70-deficient MEFs suggests that BIIB021 works, at least in part, through HSP70 in reducing the aggregation of PMP22 (Fortun et al., 2007; Rangaraju et al., 2008). This reduction in aggregation might alleviate proteasome impairment and provide functional benefits for the cells. Age-related decrease in chaperone response could then contribute to the progression of PMP22-linked neuropathies (Soti and Csermely, 2002).

Targeting of undegraded proteasome substrates to autophagy has been shown in a number of disease models, including CMT1A (Fortun et al., 2003; Minoia et al., 2014). The ability of HSP70 to recognize and bind misfolded substrates is thought to be utilized in targeting cargo to degradative pathways (Kettern et al., 2010). This activity requires CHIP, an ubiquitin-ligating enzyme, which ubiquitinates HSP70-bound substrates and depending on binding of the Bcl-2 associated athanogene (BAG) proteins, the HSP70-substrate-CHIP complex is directed either to proteasomal or autophagic degradation (Kettern et al., 2010; Minoia et al., 2014). Here, we found that under proteasomal inhibition, HSP70 reduces PMP22 aggregation partially through autophagy (Figure 4). As 3-MA specifically blocks macroautophagy and PMP22 lacks the classical KFERQ sequence present in CMA (Chaperone-mediated autophagy)-substrates (Kaushik et al., 2011), a possible involvement of CMA can be ruled out (Finn et al., 2005).

If HSP70.1/3 has a role in assisting the processing of PMP22, one may expect to see myelin-related peripheral neuropathy in HSP70.1/3 knock out animals. While rigorous studies of peripheral nerve function in such mice have not been reported, rotarod tests at 3 months of age did not reveal impairments (Okamoto et al., 2013). In agreement, analyses of PMP22 at steady-state in nerves of affected mice detected only ∼10% reduction in EndoH-resistant PMP22 as compared with Wt. It is possible that in nerves of these transgenic mice, compensatory mechanisms are activated to assure trafficking of PMP22 to the plasma membrane. For example, PMP22 interacting protein such as P0 (D'Urso et al., 1999) could promote the processing and transport of PMP22 to the plasma membrane in the absence of HSP70. Nonetheless, our *in vitro* findings under proteotoxic stress support a role for HSP70 in assisting the folding and maturation of PMP22. In agreement are data from *in vivo* studies where the ability of TrJ mice to stay on the rotarod was severely impacted in the absence of HSP70 (Okamoto et al., 2013). In that study, the authors showed increased transcript levels of ER-stress sensor proteins in nerves of the TrJ/HSP70.1/3^−/−^ mice and hypothesized such changes to be responsible for the intensified phenotype. In our cell culture model, we found no evidence for the induction of the unfolded protein response, and this was consistent in the Wt and HSP70-deficient backgrounds (Figure 6(f)). This difference could be due to the transient expression of TrJ-PMP22 in our model and the ability of MEFs to efficiently remove the misfolded TrJ-PMP22. Nevertheless, both studies support a role for HSP70.1/3 in the processing of TrJ-PMP22.

With regard to mechanism, it is unclear as to how a cytoplasmic chaperone aids in the trafficking of a tetraspan membrane protein. Our studies show partial colocalization of LAMP1^+^ vesicles with TrJ-HA3, suggesting a role for HSP70 in lysosomal degradation. However, a similar recruitment of lysosomes to TrJ-PMP22 aggregates in HSP70.1/3^−/−^ MEFs indicates that HSP70 might not be crucial for this process. Because TrJ-PMP22 is shown to be directed to the lysosomal pathway (Notterpek et al., 1997; Fortun et al., 2003), this is an expected result. On the other hand, why does the biochemistry show such significant differences in processing of TrJ-PMP22 between the genotypes when immunostaining detects a similar LAMP1 colocalization in presence and absence of HSP70? It is possible that EndoH-resistant TrJ-PMP22 is targeted for autophagy-lysosomal degradation after endocytosis from the plasma membrane (Villarroel-Campos et al., 2014). In this regard, increased levels of Rab7 and its colocalization with the mutant protein in Wt, but not in HSP701.3^−/−^ MEFs, suggests that HSP70 helps in the transport of TrJ-PMP22 from the ER to the Golgi and then to lysosomes via the endocytic Rab7 pathway. To fully understand how HSP70 influences the processing of Wt and TrJ-PMP22, pulse-chase analyses in nerve tissue from HSP70.1/3^−/−^ and HSP70.1/3^−/−^XTrJ progeny will be necessary. Nevertheless, the observed positive effects of elevated HSP70 in preventing the accumulation of undegraded proteasome substrates support further study of HSP90 inhibitors in models of PMP22-linked neuropathies.

## Summary

Our results show that the chaperone, HSP70, is critical in preventing the buildup of misfolded myelin protein 22 (PMP22). Because abnormal protein aggregates can interfere with fundamental cellular functions, enhancement of chaperones should be considered as therapy for PMP22-linked neuropathies.
